# Exploration of serum sensitive biomarkers of fatty liver in dairy cows

**DOI:** 10.1038/s41598-018-31845-0

**Published:** 2018-09-11

**Authors:** Yizhao Shen, Lianmin Chen, Wenzhu Yang, Zhonghua Wang

**Affiliations:** 1 Ruminant Nutrition and Physiology Laboratory, College of Animal Science and Technology, Shandong Agricultural University, Taian, 271018 P.R. China; 2 Agriculture and Agri-Food Canada, Lethbridge Research Centre, Alberta, T1J 4B1 Canada; 3 Department of Paediatrics & Department of Genetics, University Medical Centre Groningen, University of Groningen, Groningen, 9700 RB The Netherlands

## Abstract

Serum proteins are sensitive with diseases in dairy cows, and some of them could be used as biomarkers for fatty liver. This study aimed to explore serum biomarkers for fatty liver in dairy cows. A total of 28 early lactating dairy cows were chosen from a commercial dairy herds, liver samples were collected for determining concentration of triacylglycerol (**TAG**), and serum samples were collected for measuring fibroblast growth factor-21 (**FGF-21**), adiponectin, Lipoprotein-associated phospholipase A2 (**LP-PLA2**), and hemoglobin (**Hb**). Dairy cows were divided into fatty liver (liver TAG > 5%, wet weight) and control group (liver TAG < 5%, wet weight). Concentration of FGF-21 was greater in fatty liver cows, while the concentration of LP-PLA2 and Hb was less. The concentration of FGF-21 and total Hb had strong correlation with the liver TAG as well as good prediction power (kappa value = 0.79 and 0.58, respectively). These results suggested that the serum concentration of FGF-21 and total Hb could be potentially used as fatty liver biomarkers in lactating dairy cows.

## Introduction

Fatty liver is a common metabolic disorder in dairy cows during transition period^1^. Lactating dairy cows during transition period often suffer from negative energy balance (NEB) which leads to mobilization of body adipose tissue to meet the energy requirement. Such mobilization often results in an increase in blood concentration of non-esterified fatty acid (NEFA)^2^. The NEFA can be absorbed by liver, re-esterified to triacylglycerol (TAG), accumulated in the liver, and consequently fatty liver produced^3^. Up to 54% dairy cows have liver TAG accumulation of more than 5% of wet weight of liver during early lactation^4^. Fatty liver may decrease milk production, and lead to some clinical and subclinical diseases^1,5,6^. The fatty liver can cost dairy industry in the United States up to $60 million every year^1^.

Though fatty liver exists as common metabolic disorder for long time and its considerable economic losses for dairy industry, it still lacks effective diagnosis methods. The only credible diagnostic method is the liver biopsy. The diagnosis through biopsy is not a practical method at farm level since it needs special training and presents high risk of infection^7^. Furthermore, liver biopsy can damage liver and adversely impact cattle production and health. Therefore, there is need to develop practical and cost-effective methods for diagnosis of fatty liver.

Blood is easily to be collected, and it has the most comprehensive proteome, and thus, can be potentially used to monitor or to diagnose diseases. Many proteins have been confirmed as biomarkers in non-alcohol fatty liver disease (NAFLD) of human beings, such as fibroblast growth factor-21 (FGF-21), adiponectin (ADP), Lipoprotein-associated phospholipase A2 (LP-PLA2), hemoglobin (Hb)^8–10^. Similar to the NAFLD of human beings, fatty liver of dairy cows is also a type of disease due to excessive lipid storage in the liver, and decrease the metabolic functions of liver^1^. Therefore, this study was to explore if the confirmed biomarker for NAFLD in human beings are sensitive in dairy cows.

## Results

### Characteristics of dairy cows with different fatty liver status

Of the 28 dairy cows in our study, 15 cows had a liver TAG > 5% (wet weight basis). The calculated fatty liver incidence rate was 53.6% and the average liver TAG concentration during 22 to 35 days postpartum was 6.80%. The liver TAG concentration in fatty liver cows was greater (P < 0.01) than control cows in our study, but the background of the dairy cows including age, dry matter intake, day in milk, body weight and milk yield were not different between these two groups. Although the serum concentration of ADP was not changed, FGF-21 was greater (P < 0.01) in fatty liver cows and rest serum proteins including LP-PLA2, Hbα, Hbβ and total Hb was greater (P < 0.05) in control group (Table 1).

**Table 1:** Characteristics of dairy cows with different fatty liver status. Data are presented as means ± SD and ranges in parenthesis.

Variables	Control (n = 13)	Fatty liver (n = 15)	P
Liver triglyceride (% wet weight)	2.64 ± 0.90 (1.64–4.16)	10.41 ± 5.15 (5.80–22)	<0.01
Age (mon)	55 ± 8 (44–69)	58 ± 7 (43–66)	0.26
Dry matter intake (kg/d)	20.6 ± 2.3 (15.5–22.9)	19.4 ± 3.0 (13.4–24.5)	0.21
Day in milk (day)	29 ± 5 (21–37)	26 ± 4 (21–35)	0.06
Body weight (kg)	650 ± 53 (582–677)	668 ± 45 (603–720)	0.34
Milk yield (kg/d)	26.0 ± 1.9 (20.8–28.5)	25.6 ± 2.6 (21.9–31.7)	0.67
FGF-21 (pg/mL)	954 ± 70 (746–1040)	1112 ± 145 (971–1367)	<0.01
LP-PLA2 (μg/L)	216 ± 15 (194–255)	203 ± 9 (184–215)	0.01
ADP (μg/mL)	34.3 ± 3.6 (29.3–39.8)	32.3 ± 7.1 (23.6–51.9)	0.36
Hemoglobin α (μg/mL)	27.4 ± 1.9 (24.1–31.4)	25.6 ± 1.8 (22.5–27.8)	0.02
Hemoglobin β (μg/mL)	16.7 ± 1.4 (13.7–19.6)	15.0 ± 2.2 (12.1–19.1)	0.03
Total hemoglobin (μg/mL)	43.9 ± 2.2 (39.6–48.2)	40.8 ± 2.9 (36.2–46.7)	<0.01

Abbreviations: FGF-21, fibroblast growth factor-21; LP-PLA2, Lipoprotein-associated phospholipase A2; ADP, adiponectin.

### Pearson correlation coefficients between serum proteins and liver triglyceride

**Figure 1:**
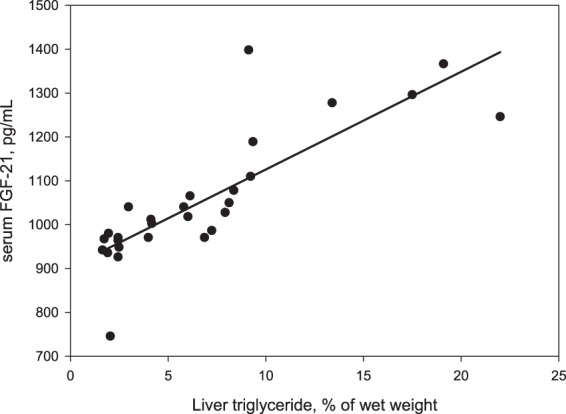
The relationship between serum fibroblast growth factor-21 (FGF-21) and liver triglyceride concentration (r = 0.74; P < 0.01).

**Figure 2:**
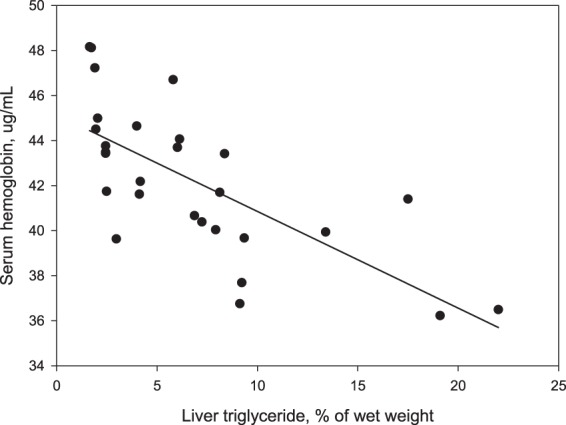
The relationship between serum hemoglobin and liver triglyceride concentration (r = −0.65; P < 0.01).

To evaluate if the serum proteins concentration were sensitive to liver TAG concentration, the Pearson correlation coefficients between serum proteins and liver TAG were calculated. A positive relationship between serum FGF-21 and liver TAG (r = 0.74; P < 0.01; Fig. 1) was detected, whereas, Hb including Hbα, Hbβ and total Hb had negative relationship with liver TAG (r = −0.57, −0.56 and −0.65, respectively; P < 0.01; Table 2 and Fig. 2). No significant relationships with liver TAG were observed in either LP-PLA2 or ADP (Table 2).

**Table 2:** Pearson correlation coefficients between serum proteins and liver triglyceride. Abbreviations: LP-PLA2, Lipoprotein-associated phospholipase A2; ADP, adiponectin.

Proteins	Pearson correlation coefficient with liver triglyceride	P
LP-PLA2	−0.27	0.17
ADP	−0.25	0.21
Hemoglobin α	−0.57	0.01
Hemoglobin β	−0.56	<0.01

### Use of serum concentration of FGF-21 and total Hb to predict fatty liver in dairy cows

**Figure 3:**
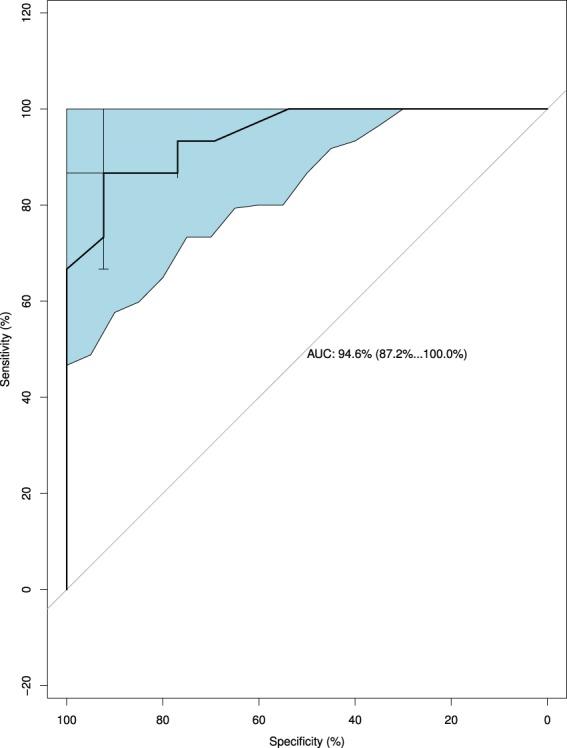
Receiver operating characteristic (ROC) curve of fibroblast growth factor-21 (FGF-21) with 95% confidence intervals. The area under curve (AUC) is 0.946 (from 0.872 to 1), and the cut-off value is 1015 pg/mL with the sensitivity of 86.7% and specificity of 92.3%.

**Figure 4:**
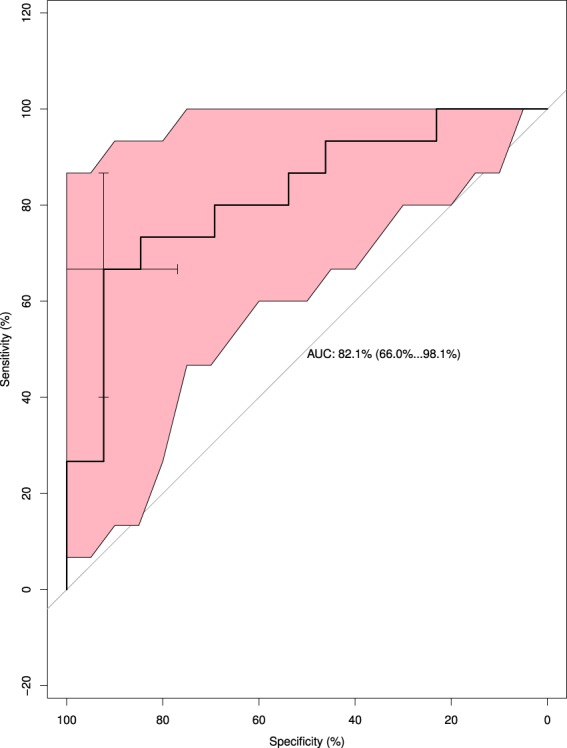
Receiver operating characteristic (ROC) curve of hemoglobin with 95% confidence intervals. The area under curve (AUC) is 0.821 (from 0.660 to 0.981), and the cut-off value is 41.5 μg/mL with the sensitivity of 92.3% and specificity of 66.7%.

The receiver operating characteristic (ROC) curve in Figs 3 and 4 indicated that both FGF-21 and total Hb can be used as biomarkers for fatty liver in dairy cows since the area under curve (AUC) of FGF-21 and total Hb were 0.946 and 0.821, respectively. The cut-off value of FGF-21 was 1015 pg/mL, with the sensitivity of 86.7% and specificity of 92.3%, while the cut-off value of total Hb was 41.5 μg/mL, with the sensitivity of 92.3% and specificity of 66.7%.

Details of each ROC curve with confidence intervals of 95% (95% CI) and the cut-off values (marked as cross bars) were shown in Figs 3 and 4.

The usefulness of FGF-21 and total Hb as predictors of fatty liver in dairy cows was further investigated (Table 3). The concentration of FGF-21 showed a strong predictive power (kappa value = 0.79; P < 0.01) with a predictive accuracy, sensitivity and specificity of 89.7, 86.7 and 92.9%, respectively. Similarly, a significant predictive power of fatty liver was also found in the serum concentration of total Hb (kappa value = 0.58; P < 0.01). The predictive accuracy, sensitivity and specificity of fatty liver using total Hb was 79.3, 72.2 and 90.9%, respectively.

**Table 3:** Kappa value, accuracy, sensitivity and specificity of identified protein in fatty liver prediction in dairy cows.

Proteins	Kappa Value	Accuracy (%)	Sensitivity (%)	Specificity (%)
FGF-21	0.79*	89.7	86.7	92.9
Total Hemoglobin	0.58*	79.3	72.2	90.9

Abbreviations: FGF-21, fibroblast growth factor-21.

*Means P < 0.01.

## Discussion

Fatty liver is a common metabolic disorder in high-producing dairy cows, the incidence could be more than 50% at postpartum^1^. In this study, the fatty liver incidence of 53.6% in cows during 22 to 35 days postpartum was in consistent with the incidence of 53.7% reported in dairy cows during 38 to 51 days postpartum^5^. Furthermore, the liver TAG concentration ranged from 1.6 to 22.0% was also similar with the 1.3 to 20.9% reported previously^4^. It suggests that the cows we chosen in this study were representative.

An interesting finding among these cows was the concentration of serum FGF-21 that was positively associated with liver TAG concentration. The FGF-21 is known as a kind of protein expressed predominantly in the liver, and plays important roles in lipid and glucose metabolism^9^. Many studies proved that FGF-21 can be a novel biomarker for fatty liver in human beings^9,11^, and showed a significant positive correlation with liver fat^12^. Similarly, the present results showed greater concentration of FGF-21 in fatty liver cows, and the positive correlation between concentration of FGF-21 and liver TAG. The expression of FGF-21 was reported to be greater with increasing of NEFA and β-hydroxybutyric acid in mice after 24 h fasting^13^, and was regulated directly by peroxisome proliferator-activated receptor α (PPARα)^14^. Fatty liver in dairy cows were induced by NEB, a similar condition like the fasted model, showed a similar increase of NEFA and β-hydroxybutyric acid^2^. Both NEB and NEFA could regulate the expression of PPARα^15^, which would partially explain the increase of FGF-21 with fatty liver group cows in this study. Schlegel *et al*. (2013) reported an increased the expression of FGF-21 in the liver at the first week postpartum, where it is the highest incidence of fatty liver in dairy cows, and showed a positive correlation^16^. Schoenberg *et al*. (2011) also suggested that use of FGF-21 as a potential biomarker for fatty liver in dairy cows based on their observation of positive correlation between plasma FGF-21 and liver TAG in multiparous Holstein cows during transition period^17^. Therefore, both previous studies and the results with FGF-21 in this study including the positive association with liver TAG and strong prediction power of liver TAG illustrated that FGF-21 could be a potential biomarker for predicting fatty liver in dairy cows.

However, though the concentration of LP-PLA2 differed between fatty liver and control group, no correlation of LP-PLA2 and liver TAG was detected. It suggested that the concentration of serum LP-PLA2 may not be a biomarker for fatty liver in dairy cows. The LP-PLA2 is a protein produced by a wide range of inflammatory and non-inflammatory cells^18^. Recently, a study with 57 NAFLD patients and 38 healthy people found that the level of LP-PLA2 was greater in patients with fatty liver, and the concentration of LP-PLA2 was strongly associated to histological steatosis scores in patients with NAFLD^8^. The LP-PLA2 is usually used as a biomarker for diagnosing cardiovascular disease (CVD)^19^, and many studies in human beings have proven that CVD and NAFLD were associated each other. Absence of reporting CVD in dairy cows may partly explain why the serum concentration of LP-PLA2 in fatty liver cows did not show the same variation with that in human beings. Previous study showed positive correlation of serum LP-PLA2 with body mass index and waist circumference, but no correlation between TAG and alanine aminotransferase were observed^8^. It appears that the concentration of serum LP-PLA2 may not be influenced by the fatty liver in dairy cows, and thus cannot be a biomarker for the fatty liver.

Another interesting finding was that the concentration of serum Hbα, Hbβ and total Hb were less in fatty liver cows compared with control cows and negatively correlated with the concentration of liver TAG, indicated that the concentration of serum Hb may be a biomarker for fatty liver in dairy cows. However, in the current study with dairy cows, the negative correlations between serum Hb and liver TAG contrasted to the positive correlations reported in previous studies with humans^20–22^. The discrepancy could be due to the difference in CVD between animals and human beings. As discussed previously, NAFLD patients are usually associated with CVD, led to increasing blood viscosity and reducing blood flow and as a result, the capacity of Hb to transport oxygen reduced. The increased Hb concentration in NAFLD patients could be resulted from the hepatic hypoxia^23^. Previous studies evidenced hemoglobin as biomarker of CVD^24^. However, for dairy cows, CVD was not reported during fatty liver, and it does not need to improve the capacity to transport oxygen by enhancing the concentration of Hb. In addition, the difference in concentration of serum Hb, and the negative correlation with liver TAG can be due to the variation of the expression of heme-oxygenase, which is an enzyme being able to degrade the Hb, thus its activity may be negatively associated with obese status. Trak-Smayra *et al*.^20^ also found that the body weight reduction lead to the decreasing of Hb using a proteomics based method^20^. The NAFLD patients are often associated with larger body mass index and waist circumference^8^, while cows with fatty liver usually suffer from lower BCS and more severe loss of BCS^5^. It indicates that the activity of heme-oxygenase should be less in NAFLD patients, but greater in fatty liver cows, and thus lead to the concentration of Hb increasing in human beings but decreasing in dairy cows. Otherwise, some other stress that is closely related to fatty liver in dairy cows like calving, ketosis or mastitis^6^, also proved to decrease the concentration of Hb by previous studies^25–27^. In current study, the strong association with liver TAG, great AUC as well as the significant kappa value also indicated that the concentration of Hb may be sensitive to fatty liver in dairy cows, concluded that Hb can be another potential biomarker for fatty liver.

Adiponectin is a protein mainly secreted by adipose tissue, plays important roles in glucose and fatty acid metabolisms^28^. Recent studies found that the concentration of serum ADP was significantly (*P* < 0.05) lower in NAFLD patient^29^. Louthan *et al*. found negative correlation of serum concentration of ADP with body mass index, insulin, glucose and ALT in human beings^30^. However, in dairy cows the correlation of the concentration of ADP with blood insulin, glucose or NEFA was not detected^31^. In fact, the function of ADP was regulated not only by its serum concentration, but also by the expression of receptor^31^. The lack of difference in serum ADP between control and fatty liver cows in our study suggested that the concentration of serum ADP may not be a sensitive indicator of fatty liver in dairy cows.

In conclusion, serum concentrations of FGF-21, LP-PLA_2_, Hbα, Hbβ and total Hb differed between fatty liver and control dairy cows. The concentration of serum FGF-21 and total Hb were highly or moderately correlated with the concentration of liver TAG, and had good power to predict liver TAG concentration. These results indicate that the concentration of serum FGF-21 and total Hb could be potentially developed as biomarkers to detect fatty liver in dairy cows. Further studies using large number of dairy cows were warranted to confirm this finding.

## Materials and Methods

### Sample collection

This study was conducted by following the standard protocol established by the College of Animal Science and Technology, Shandong Agricultural University, Shandong, China, and approved by the Animal Care and Use Committee of Shandong Agriculture University. Twenty-eight lactating Holstein dairy cows (mean ± SD; Day in milk: 28 ± 4 days; Age: 56 ± 7 month; Body weight: 660 ± 44 kg) in similar status were selected from a commercial dairy herd in Taian, Shandong, China. All cows were fed a total mixed ration consisting of 55.4% concentrate and 44.6% forage (alfalfa and oat hay; dry matter [DM] basis) with 17.7% crude protein. Cows were fed and milked 3 times daily at 0600 h, 1400 h and 2200 h, respectively, with 40%, 30 and 30% of total diet offered. Refusals were collected once daily before the morning feeding. Cows were housed in individual tie stalls, and had free access to water. All cows had similar calving date, milk yield (25.6 ± 2.1 kg/d), DM intake (19.92 ± 3.12 kg/d) and had no clinical diseases.

Cows were weighed twice at 3 days before the sampling and the sampling day, respectively. Daily feed intake was calculated as the difference between feed offered and refusals during 7 days before sampling. Blood and liver samples were collected at 0600 h before morning feeding. Liver biopsy was conducted at about 10 cm above the point between the 11^th^ and 12^th^ ribs on the line between the hip and right elbow. About 1 g liver sample was collected by a trained veterinarian, using a liver biopsy needle. The collected liver samples were stored at −80 °C until analysis. Blood samples were collected via jugular vein, using a 10 mL evacuated tube (Becton Dickinsn, Franklin Lake, NJ, USA) without any additive, clotted within room temperature for 30 min, and centrifuged at 1 500 × g for 20 min at 4 °C to obtain serum. The serum was stored at −20 °C until analysis.

### Laboratory Analyses

Liver TAG was extracted using the mixture of hexane and isopropanol, as described by Starke *et al*.^7^. The concentration of TAG was determined using commercial TAG kits (Jiancheng, Nanjing, China). The cows were divided into control group (n = 13) or fatty liver group (n = 15) according to the standard of 5% liver TAG (wet weight basis). Serum FGF-21 and LP-PLA2 were measured using R&D Human FGF-21 ELISA kit and R&D Human LP-PLA2 (R&D Systems Inc. Minneapolis, MN, USA)^32^, serum Hbα, Hbβ and ADP was measured using a CUSABIO Bovine Hbα, Hbβ and ADP ELISA kit (CUSABIO Biotech Co. Ltd. Wuhan, China)^33^.

### Statistical Analysis

The concentration of liver TAG and serum proteins and patient data were analyzed using PROC MEANS of SAS (ver. 9.3, SAS Inst., Cary, NC). The Pearson correlation coefficient between the concentration of serum proteins and liver TAG was analyzed using PROC CORR of SAS. Treatment effects were declared significant at *P* ≤ 0.05. For further evaluate if total Hb and FGF-21 can be used as predictors in fatty liver, receiver operating characteristic (ROC) analysis was carried out by using *R* (version 3.4.3) package *pROC* with function *roc*. We also compared the area under curve (AUC) between different ROC curves by using function *roc*.*test*. The kappa value, accuracy, sensitivity and specificity of serum FGF-21 and total Hb to predict liver TAG were calculated in excel 2011.

### Ethical approval and informed consent

The study was conducted by following the standard protocol established by the College of Animal Science and Technology, Shandong Agricultural University, Shandong, China, and approved by the Animal Care and Use Committee of Shandong Agriculture University.
